# Cerebrovascular, Cognitive and Cardiac Benefits of SGLT2 Inhibitors Therapy in Patients with Atrial Fibrillation and Type 2 Diabetes Mellitus: Results from a Global Federated Health Network Analysis

**DOI:** 10.3390/jcm12082814

**Published:** 2023-04-11

**Authors:** Riccardo Proietti, José Miguel Rivera-Caravaca, Raquel López-Gálvez, Stephanie L. Harrison, Francisco Marín, Paula Underhill, Eduard Shantsila, Garry McDowell, Manlio Vinciguerra, Rhys Davies, Clarissa Giebel, Deirdre A. Lane, Gregory Y. H. Lip

**Affiliations:** 1 Liverpool Centre for Cardiovascular Science at University of Liverpool, Liverpool John Moores University and Liverpool Heart & Chest Hospital, Liverpool L8 7TX, UK; 2 Department of Cardiovascular and Metabolic Medicine, Institute of Life Course and Medical Sciences, University of Liverpool, Liverpool L8 7TX, UK; 3 Department of Cardiology, Hospital Clínico Universitario Virgen de la Arrixaca, University of Murcia, Instituto Murciano de Investigación Biosanitaria (IMIB-Arrixaca), CIBERCV, 30120 Murcia, Spain; 4 School of Nursing, University of Murcia, 30120 Murcia, Spain; 5 TriNetX LLC, London EC3V 4AB, UK; 6 Department of Primary Care and Mental Health, University of Liverpool, Liverpool L8 7TX, UK; 7 The Walton Centre NHS Foundation Trust, Lower Lane, Liverpool L9 7LJ, UK; 8 NIHR Applied Research Collaboration North West Coast, Liverpool L8 7TX, UK

**Keywords:** SGLT2, atrial fibrillation, dementia, stroke

## Abstract

Background: Sodium-glucose co-transporter 2 inhibitors (SGLT2i) are effective anti-diabetic drugs improving cardiovascular outcomes in type 2 diabetes mellitus (T2DM) patients. This study investigated cardiovascular, cerebrovascular and cognitive outcomes of SGLT2i therapy in patients with atrial fibrillation (AF) and T2DM. Methods: Observational study using TriNetX, a global health research network of anonymised electronic medical records from real-world patients between January 2018 and December 2019. The network includes healthcare organisations globally but predominately in the United States. AF patients (ICD-10-CM code: I48) with T2DM were divided according to SGLT2i use or not, and balanced using propensity score matching (PSM). Patients were followed-up for 3-years. The primary endpoints were ischaemic stroke/transient ischemic attack (TIA), intracranial haemorrhage (ICH), and incident dementia. Secondary endpoints were incident heart failure and mortality. Results: We identified 89,356 AF patients with T2DM of which 5061 (5.7%) were taking a SGLT2i. After PSM, 5049 patients (mean age 66.7 ± 10.6 years; 28.9% female) were included in each group. At 3-years follow-up, the risk of ischaemic stroke/TIA was higher in patients not receiving SGLT2i (HR 1.12, 95% CI 1.01–1.24) and for ICH (HR 1.57, 95% CI 1.25–1.99) and incident dementia (HR 1.66, 95% CI 1.30–2.12). Incident heart failure (HR 1.50, 95% CI 1.34–1.68) and mortality (HR 1.77, 95% CI 1.58–1.99) risks were increased in AF patients not receiving SGLT2i. Conclusions: In our large ‘real world’ analysis of patients with concomitant AF and T2DM, SGLT2i reduced the risk of cerebrovascular events, incident dementia, heart failure and death.

## 1. Introduction

The sodium-glucose co-transporter 2 inhibitors (SGLT2i) are new effective anti-diabetic drugs, which act by inhibiting glucose and sodium reabsorption at the level of proximal convoluted tubule of the nephron, and improving glycemic control in patients with type 2 diabetes mellitus (T2DM) [1]. Currently, four SGLT2i are approved by the European Medicines Agency and the Food and Drug Administration and available to prescribe: canagliflozin, dapagliflozin, empagliflozin and ertugliflozin [1].

Two features of SGLT2i are of particular interest: (i) a mechanism of action not linked to insulin secretion, which makes the detrimental occurrence of hypoglycemia less likely compared to other anti-diabetic drug classes; and (ii) cardiovascular and renal protective effects independent of its’ glycemic control and noted early after initiation of the therapy, suggesting mechanisms of action beyond blood glucose lowering [1,2].

Three randomized clinical trials, the Empagliflozin Cardiovascular Outcome Event Trial in Type 2 Diabetes Mellitus Patients (EMPA-REG OUTCOME) [3], CANagliflozin cardioVascular Assessment Study (CANVAS) [4], and Dapagliflozin Effect on Cardiovascular Events–Thrombolysis in Myocardial Infarction 58 (DECLARE-TIMI 58) trial [5], have reported that SGLT2i improved cardiovascular outcomes in patients with T2DM, including a reduced risk of cardiovascular death and hospitalization for heart failure. In expanding the applicability of SGLT2i therapy, the ‘EMPagliflozin outcomE tRial in patients with chrOnic heaRt failure (EMPEROR-reduced)’ investigated 3730 patients with reduced ejection fraction (<40%) of whom 49% had T2DM, and showed a reduction of the combined primary endpoints (cardiovascular death and heart failure hospitalization) in the empagliflozin group compared to placebo (19.4% vs. 24.7% respectively; hazard ratio (HR) 0.76, 95% confidence interval (CI) 0.67–0.87; *p* < 0.0001). The ‘Empagliflozin Outcome Trial in Patients with Chronic Heart Failure with Preserved Ejection Fraction (EMPEROR-Preserved)’ showed that empagliflozin was superior to placebo in improving heart failure outcomes (cardiovascular death and hospitalization) among patients with symptomatic stable heart failure and preserved ejection fraction (>40%), irrespective of diabetes status (HR 0.79, 95% CI 0.69–0.90, *p* < 0.001).

The Canagliflozin and Renal Events in Diabetes with Established Nephropathy Clinical Evaluation (CREDENCE) trial [6] testing the efficacy of SGLT2i in patients with T2DM and evidence of albuminuric chronic kidney disease showed a reduction in the risk of progression toward end-stage renal failure (HR 0.68, 95% CI 0.54–0.86; *p* = 0.002). A sub-analysis of the CREDENCE trial suggested that there may be a potential effect of SGLT2i on ischaemic stroke prevention and reduction in non-traumatic brain haemorrhages [7]. More recently, a sub-analysis of the DECLARE-TIMI 58 trial found a decrease in new episodes of atrial fibrillation (AF) or atrial flutter (AFl) with SGLT2i independent from the prior history of AF, atherosclerotic cardiovascular disease or heart failure [8].

Together these data lead to the hypothesis that SGLT2i may be beneficial in patients with AF which are at risk of developing incident comorbidities including heart failure and dementia. This is further supported by the anti-inflammatory, metabolic and neuro-modulatory actions of this class of medications [2]. Nonetheless, there is limited evidence from large-scale datasets on whether these trial data translate into real-world clinical practice.

Therefore, in this study we investigated the effect of SGLT2i therapy in reducing the occurrence and risk of adverse cerebrovascular cardiovascular, and cognitive outcomes using a cohort of patients with AF and T2DM.

## 2. Materials and Methods

This is an observational and retrospective study using TriNetX, a global federated health research network with real-time updates of anonymised electronic medical records (EMRs). The network includes healthcare organisations (HCOs, academic medical centres, specialty physician practices and community hospitals) with data for >85 million real-world patients, predominately based in the United States. In brief, the TriNetX research network database encompasses anonymized EMRs of patients registered with the network and has information on patient demographics, clinical details including diagnoses (using International Classification of Diseases, Tenth Revision, Clinical Modification (ICD-10-CM) codes), medications and investigations as well as any procedures, from settings such as general practice surgeries, community and secondary hospitals. To comply with legal frameworks and ethical guidelines guarding against data re-identification, the identity of participating HCOs and their individual contribution to each dataset are not disclosed. As a federated research network, studies using the TriNetX health research network do not require ethical approval as no patient is identifiable.

For the present study, the TriNetX research network was searched for the inclusion of patients from 1 January 2018 to 31 December 2019. To be included in the study, all patients were aged ≥18 years with a diagnosis of AF (ICD-10-CM code: I48) and diabetes mellitus (ICD-10-CM code: E08-E13) during this period, and received oral anticoagulation therapy either with a vitamin K antagonist (VKA) or a non-VKA oral anticoagulant (NOAC). Patients were divided into two groups according to SGLT2i use (empagliflozin, dapagliflozin, or canagliflozin). In the group of patients on SGLT2i, only prevalent SGLT2i users were considered. Patients not receiving SGLT2i at inclusion, who were subsequently prescribed it, were censored when the drug was initiated. Patients with chronic rheumatic heart diseases (ICD-10-CM code I05-I09), acute rheumatic fever (ICD-10-CM code I00–I02) or prosthetic heart valves (ICD-10-CM code Z95.2) were excluded, with no other exclusion criteria.

We collected data on baseline demographics, ethnicity, comorbidities (e.g., hypertension, coronary artery disease, heart failure, cerebrovascular disease, peripheral vascular disease, hyperlipidemia, overweight/obesity, chronic obstructive pulmonary disease (COPD), renal impairment, diseases of the nervous system, diseases of liver and cancer), and medication use (e.g., anticoagulants, antiplatelets, beta-blockers, calcium channel blockers, antiarrhythmics, angiotensin-converting enzyme inhibitors, angiotensin II inhibitors, antilipemic agents, diuretics, and blood glucose regulation agents).

We used ICD-10-CM codes to identify relevant diagnoses and the Anatomic Therapeutic Chemical (ATC) codes to identify pharmacotherapy. The searches were run in TriNetX on 3 May 2022. At the time of the search, there were 58 participating HCOs within the TriNetX research network.

### 2.1. Follow-Up and Clinical Outcomes

All patients were followed-up for three years. The primary endpoints were ischaemic stroke/transient ischemic attack (TIA) (ICD-10-CM codes: G45 or I63), intracranial haemorrhage (ICH, ICD-10-CM codes: I60, I61 or I62), and incident dementia (either vascular dementia, Alzheimer’s disease or unspecified dementia, i.e., ICD-10-CM codes: F01, F02, F03 or G30). Secondary end-points were incident heart failure (ICD-10-CM code: I50) and all-cause mortality. We used ICD-10-CM codes to identify the primary and secondary endpoints reported in the EMRs through the TriNetX platform.

### 2.2. Statistical Analysis

Continuous variables (age) were expressed as mean and standard deviation (SD), and tested for differences with independent-sample *t* test. Categorical variables (sex, ethnicity, comorbidities, and pharmacological therapy) were expressed as absolute frequencies and percentages, and tested for differences with chi-squared test. The TriNetX platform was used to run 1:1 propensity score matching (PSM) using logistic regression. The platform uses ‘greedy nearest-neighbour matching’ with a caliper of 0.1 pooled standard deviations and difference between propensity scores ≤0.1. Covariate balance between groups was assessed using standardised mean differences (SMDs). Any baseline characteristic with a SMD between cohorts <0.1 is considered well-matched [9].

HR and 95% CI were calculated following PSM, and displayed as Kaplan-Meier survival curves with log-rank tests. No imputations were made for missing data. Two-sided *p*-values < 0.05 were accepted as statistically significant. Statistical analysis was performed using the TriNetX Analytics function in the online research platform.

## 3. Results

The study identified 89,356 patients with AF and T2DM, and of these, 5061 (5.7%) patients were taking a SGLT2i. Table 1 summarises the baseline characteristics of patients with and without SGLT2i therapy. Prior to PSM, patients receiving SGLT2i were likely be male, not Hispanic or Latino, and suffered from ischemic heart disease, hyperlipidemia, and overweight/obesity. On the contrary, patients on SGLT2i presented lower prevalence of cerebrovascular disease, COPD, and renal disease, and were more frequently treated with NOACs instead of VKAs. These differences were attenuated after PSM and both cohorts, each including 5049 patients, were balanced.

### 3.1. Cerebrovascular Events and Incident Dementia According to SGLT2i Therapy

**Figure 1:**
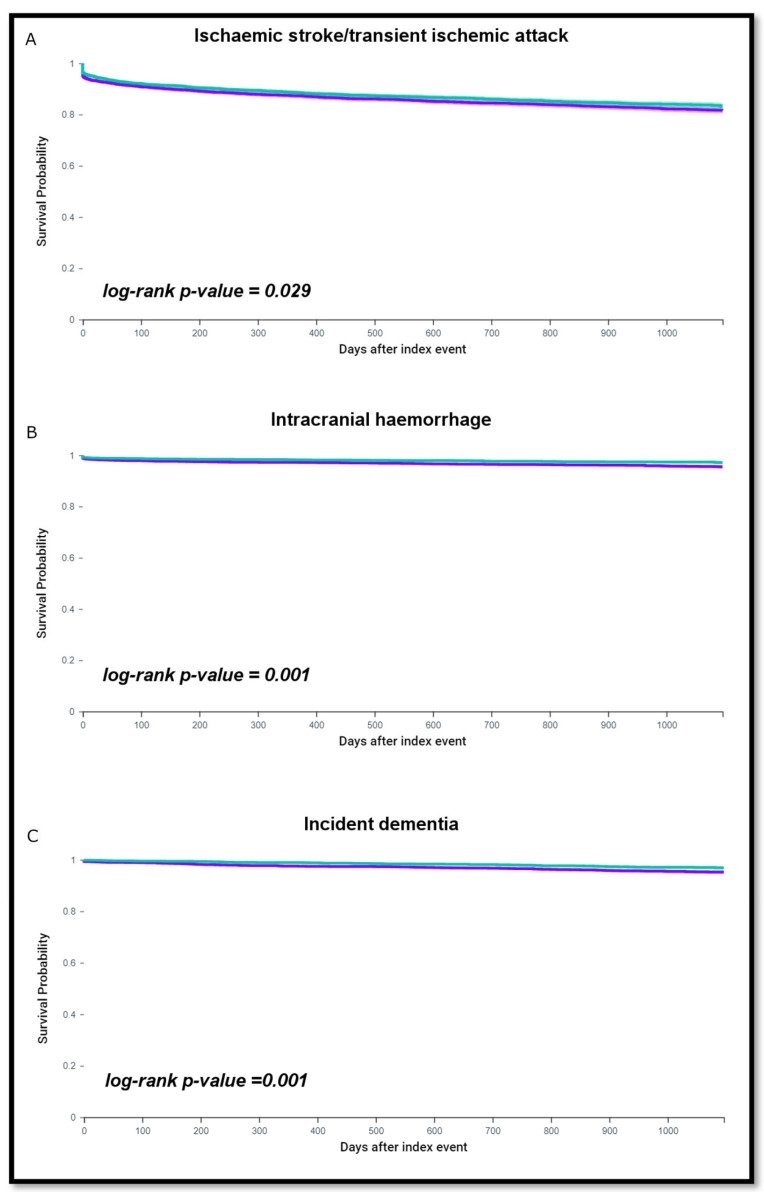
Kaplan–Meier curves showing survival free from ischemic stroke/TIA (**A**), ICH (**B**) and dementia (**C**) in patients on SGLT2 therapy versus without.

After a follow-up of three years, 767 (15.2%) patients without SGLT2i therapy and 693 (13.7%) patients on SGLT2i therapy had an ischemic stroke or TIA. The risk of ischemic stroke/TIA was increased in patients without SGLT2i compared to those taking SGLT2i (HR 1.12, 95% CI 1.01–1.24, log-rank *p* = 0.029) (Figure 1A). In addition, 183 (3.6%) patients without SGLT2i and 115 (2.3%) taking SGLT2i had an ICH. The risk of ICH was greater in AF patients without SGLT2i therapy (HR 1.57, 95% CI 1.25–1.99; log-rank *p* = 0.001), (Figure 1B).

During the 3-year follow up, 174 patients (3.6%) without SGLT2i therapy and 104 (2.1%) on SGLT2i therapy had a diagnosis of incident dementia. The risk of dementia was higher among patients without SGLT2i therapy (HR 1.66, 95% CI 1.30–2.12; log-rank *p* = 0.001), as shown in the survival analysis (Figure 1C).

### 3.2. Incident Heart Failure and Mortality

During follow-up, 736 (24.3%) AF patients without SGLT2i and 496 (16.9%) patients on SGLT2i had incident heart failure. Survival free from incident heart failure was lower in patients using SGLT2i compared to those not receiving a SGLT2i (HR 1.50, 95% CI 1.34–1.68; log-rank *p* < 0.001). Mortality risk was higher among patients not on SGLT2i (HR 1.77, 95% CI 1.58–1.99; log rank *p* < 0.001).

## 4. Discussion

The main findings of our analysis demonstrate that SGLT2i therapy is associated with a reduced incidence of cerebrovascular events and dementia in patients with AF and T2DM. In addition, there was a decrease in heart failure episodes and improved survival in patients with AF and T2DM treated with SGLT2i.

Given the high prevalence of AF and dementia worldwide, in particular among older people, our finding of a decreased incidence of dementia in the cohort of patients with AF treated with SGLT2i is important. Numerous observational studies over the past 10 years, including two meta-analyses, [10,11] have shown that AF is associated with cognitive impairment and dementia, even in the absence of clinically overt previous stroke. Patients with AF are also exposed to a significant risk of Alzheimer’s disease [12]. This finding identifies a more complex interaction between AF and cognitive decline in which vascular and degenerative mechanisms, including cerebral amyloid deposition, co-exist and interact. Recently a novel SGLT2i mechanistic theory linking changes in glucose, free fatty acids (FFA) and AA metabolism to improvements in mitochondrial functioning, was proposed [13]. The SGLT2i drugs promote the loss of glucose in the urine, which can impact the mTOR signalling pathway and modulate AMPK activity; and both molecular pathways play a role in cerebral amyloid deposition and development of Alzheimer’s disease [14]. Finally, in patients with non-alcoholic fatty liver disease, treatment with SGLT2i improves neural mitochondrial function as indicated by increased levels of circulating *n*-acetyl aspartate (NAA), a biomarker of neural mitochondrial viability [15]. Depletion of NAA, as occurs in Alzheimer’s disease, may also play a direct role in promoting amyloid deposition [15]. One ongoing pilot study is evaluating the impact of dapagliflozin treatment on patients with Alzheimer’s disease (NCT03801642). Considering the link between vascular amyloid angiopathy and Alzheimer’s disease related amyloidosis, it may be hypothesized that SGLT2i may be effective in modifying the risk of dementia in patients with AF.

Sub-analysis of the major randomised controlled trials (RCTs) and a subsequent meta-analysis have proposed a possible benefit of SGLT2i in reducing the incidence of stroke; the effect seems to correlate with baseline kidney function being more evident for those with low estimated glomerular filtration rate [7,16]. Increasing evidence also suggests anti-arrhythmic effects of SGLT2i with a reduced incidence of newly diagnosed AF or AF burden in patients with already diagnosed AF [8,17]. Such anti-arrhythmic properties preventing the development of new incident AF/AFL, may underline the suggested impact of SGLT2i therapy on reducing stroke incidence. Indeed, the risk of AF is increased in patients with chronic kidney disease, thereby justifying the benefit in this group of patients. In our study, the two cohorts identified were adjusted for chronic kidney disease after PSM, which may confirm a benefit from SGLT2i therapy independently from baseline renal function. In addition, various vascular and systemic effects of SGLT2i have been described beside the lowering of blood glucose, which can help decrease the risk of cerebrovascular events, including modulation of anti-inflammatory effects with a decrease in epicardial fat, leptin and TNFα production, and improvements in vascular function with a decrease in blood pressure, vascular stiffness and uric acid levels [18].

Of note, our analysis shows a benefit of SGLT2i on reducing non-traumatic ICH.

Our findings report an absolute difference in means of approximately 1.3% and the risk of ICH was 1.57-fold higher in AF patients without SGLT2i therapy, which may underline important clinical implication. Indeed, currently there are no drugs that have shown effect in preventing ICH.

Large prospective studies have shown that both hypoglycaemic and hyperglycaemic episode are associated with ICH [19]. The SGLT2i have a mechanism of action not linked to insulin secretion, which makes the detrimental occurrence of hypoglycemia less likely compared to other classes of anti-diabetic drugs and also allows the achievement of steady levels of blood glucose. This feature along with the aforementioned actions on blood pressure lowering and improvement in endothelial function and vascular stiffness may explain the possible benefits of SGLT2i on ICH.

In our analysis, the benefits of SGLT2i on the risk of heart failure and overall survival among patients with AF were evident from the early stages of follow-up. Indeed, large RCTs of SGLT2i have demonstrated improved cardiovascular outcomes in patients with T2DM, including a reduced risk of cardiovascular death and hospitalisations for heart failure [3,4,5]. Both the DAPA-HF [20] and EMPEROR-Reduced trial [21] have shown that in patients with heart failure and reduced ejection fraction, the benefit of SGLT2i are independent of diabetes mellitus. The EMPEROR-Preserved trial [22] has further extended this finding showing similar effects in terms of reduction of cardiovascular morbidity and mortality in patients with and without T2DM who have concomitant heart failure with preserved ejection fraction.

### Limitations

Several limitations should be considered when interpreting the results of the current study. First our analysis was a retrospective study performed on EMR, and from this dose and duration of SGTL2i treatment could not be ascertained. Accordingly, a comparison between different SGLT2i and dose could not be performed. It is also possible that some changes to prescriptions may have been made outside of HCOs and not been captured within the TriNetX health research network. Furthermore, in our analysis, the cohorts were matched for factors including age, sex, ethnicity and co-morbidities, but residual confounding may still be present and some health conditions may be underreported in EMRs. Finally, an assessment of socio-economic and genetic factors biasing the associations between AF and dementia could not be performed.

## 5. Conclusions

In our large ‘real-world’ analysis of patients with concomitant AF and T2DM, SGLT2i therapy significantly reduced the risk of cerebrovascular events and incident dementia, heart failure and mortality. Considering the epidemiological relevance of the link between AF and dementia, the possible benefit of the metabolic effect of SGLT2i in preventing cognitive deterioration in this group of patients requires testing in a prospective study.

## Figures and Tables

**Table 1:** Comparison of baseline clinical characteristics of the study cohort before and after propensity score matching.

	Initial Populations						Propensity Score Matched Populations					
*n* (%)	Patients not on SGLT2 Inhibitors *n* = 84,295		Patients on SGLT2 Inhibitors *n* = 5061		*p*-Value	SMD	Patients not on SGLT2 Inhibitors *n* = 5049		Patients on SGLT2 Inhibitors *n* = 5049		*p*-Value	SMD
Age (years), mean (SD)	71.8	11.3	66.6	9.92	<0.001	0.490	66.7	11.3	66.7	9.91	0.727	0.007
Female sex, *n* (%)	33,708	33.99	1415	27.96	<0.001	0.256	1445	28.62	1413	27.99	0.480	0.014
**Ethnicity, *n* (%)**												
Hispanic or Latino	4832	5.73	309	6.11	0.268	0.016	297	5.88	309	6.12	0.615	0.010
Not Hispanic or Latino	65,725	77.97	4171	82.42	<0.001	0.112	4190	82.99	4161	82.41	0.445	0.015
**Comorbidities, *n* (%)**												
Hypertension	77,654	92.12	4761	94.07	0.457	0.077	4728	93.64	4749	94.06	0.384	0.017
Ischemic heart disease	45,790	54.32	3004	59.36	<0.001	0.102	3595	71.20	3593	71.16	0.965	0.001
Heart failure	38,334	45.48	2243	44.32	0.108	0.023	2941	58.25	2967	58.76	0.599	0.010
Peripheral vascular disease	12,778	15.16	735	14.52	0.220	0.018	842	16.68	832	16.48	0.789	0.005
Hyperlipidemia	64,318	76.30	4388	86.70	<0.001	0.270	4365	86.45	4376	86.67	0.748	0.006
Cerebrovascular disease	23,948	28.41	1191	23.53	<0.001	0.111	1163	23.03	1190	23.57	0.525	0.013
Cerebral infarction	13,832	16.41	711	14.05	<0.000	0.066	811	16.06	794	15.73	0.644	0.009
Pulmonary embolism	5752	6.82	325	6.42	0.270	0.016	783	15.51	734	14.54	0.172	0.027
Other venous embolism and thrombosis	14,134	16.77	834	16.48	0.594	0.008	1163	23.03	1190	23.57	0.525	0.013
Overweight/obesity	35,938	42.63	2976	58.80	<0.001	0.328	2978	58.98	2993	59.28	0.761	0.006
Chronic obstructive pulmonary disease	20,224	23.99	950	18.77	<0.001	0.128	1872	37.08	1862	36.88	0.837	0.004
Acute kidney failure and chronic kidney disease	40,477	48.02	1866	36.87	<0.001	0.227	2118	41.95	2130	42.19	0.809	0.005
Diseases of the nervous system	57,720	68.47	3727	73.64	<0.001	0.114	3684	72.97	3717	73.62	0.458	0.015
Diseases of liver	10,910	12.94	836	16.52	<0.001	0.101	957	18.95	948	18.78	0.819	0.005
Neoplasms	33,229	39.42	2136	42.21	<0.001	0.057	2272	45.00	2236	44.29	0.471	0.014
**Pharmacological therapy, *n* (%)**												
Beta blockers	68,668	81.46	4466	88.24	<0.000	0.190	4474	88.61	4456	88.26	0.575	0.011
ACE inhibitors	40,021	47.48	3111	61.47	<0.001	0.284	3183	63.04	3103	61.46	0.101	0.033
Angiotensin II inhibitors	24,733	29.34	2038	40.27	<0.001	0.231	2018	39.97	2030	40.21	0.807	0.005
Antilipemic agents	63,394	75.21	4526	89.43	<0.000	0.379	4587	90.85	4514	89.40	0.015	0.048
Calcium channel blockers	45,152	53.56	2800	55.33	0015	0.035	2840	56.25	2791	55.28	0.326	0.020
Diuretics	58,526	69.43	3701	73.13	0.027	0.082	3724	73.76	3692	73.12	0.471	0.014
Antiarrhythmics	49,402	58.61	3385	66.88	<0.001	0.172	3339	66.13	3376	66.87	0.435	0.016
Antiplatelets	52,170	61.89	3510	69.35	<0.001	0.158	3550	70.31	3499	69.30	0.269	0.022
**Oral anticoagulants**												
Vitamin K antagonists	52,399	62.16	2852	56.35	<0.001	0.118	2828	56.01	2847	56.39	0.703	0.008
Non-vitamin K antagonist oral anticoagulants	31,896	37.84	2209	43.65	<0.001	0.165	2221	43.99	2202	43.61	0.588	0.011
**Blood glucose regulation agents**												
Metformin	34,607	41.06	3970	78.44	<0.001	0.825	4021	79.64	3958	78.39	0.124	0.031
Insulin	49,801	59.08	3622	71.57	<0.001	0.265	3632	71.94	3612	71.54	0.658	0.009
Glipizide	11,647	13.82	1325	26.18	<0.001	0.313	1309	25.93	1318	26.10	0.838	0.004
Sitagliptin	7189	8.53	1342	26.52	<0.001	0.487	1268	25.11	1330	26.34	0.158	0.028
Glimepiride	7615	9.03	1010	19.96	<0.001	0.314	972	19.25	1.003	19.87	0.437	0.015
Pioglitazone	3341	3.96	560	11.07	<0.001	0.272	557	11.03	554	10.97	0.924	0.002
Linagliptin	1973	2.34	429	8.48	<0.001	0.274	397	7.86	423	8.38	0.344	0.019
Glyburide	3221	3.82	394	7.79	<0.001	0.170	384	7.61	392	7.76	0.765	0.006
Saxagliptin	616	0.73	138	2.73	<0.001	0.154	132	2.61	135	2.67	0.852	0.004
Repaglinide	679	0.81	79	1.56	1.258	0.070	70	1.39	78	1.55	0.508	0.013
Nateglinide	373	0.44	52	1.03	0.042	0.069	53	1.05	52	1.03	0.922	0.002
Rosiglitazone	475	0.56	44	0.87	0.005	0.036	45	0.89	44	0.87	0.915	0.002
Acarbose	270	0.32	37	0.73	<0.001	0.057	31	0.61	36	0.71	0.540	0.012

ACE = Angiotensin − converting enzyme SMD = Standardized Mean Difference.

## Data Availability

The data that support the findings of this study are available from TriNetX. To gain access to the data, a request can be made to TriNetX (https://live.trinetx.com, accessed on 20 March 2023), but costs may be incurred, and a data sharing agreement is needed.
